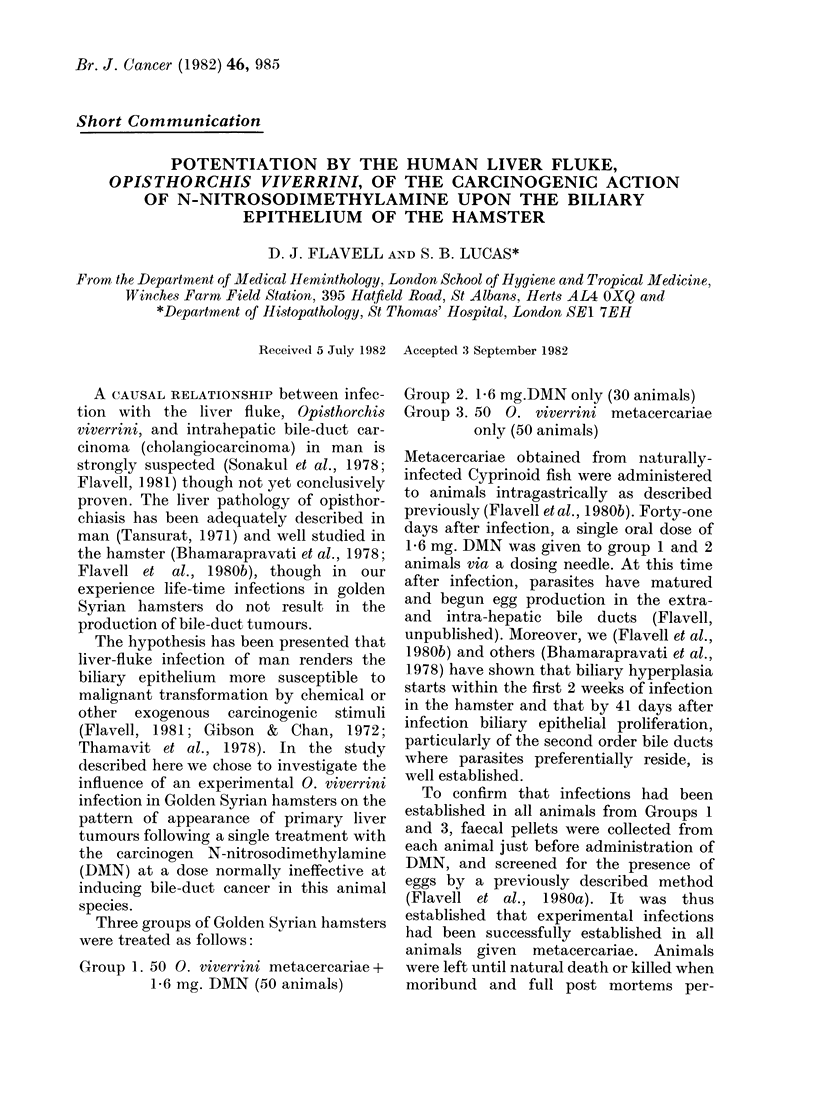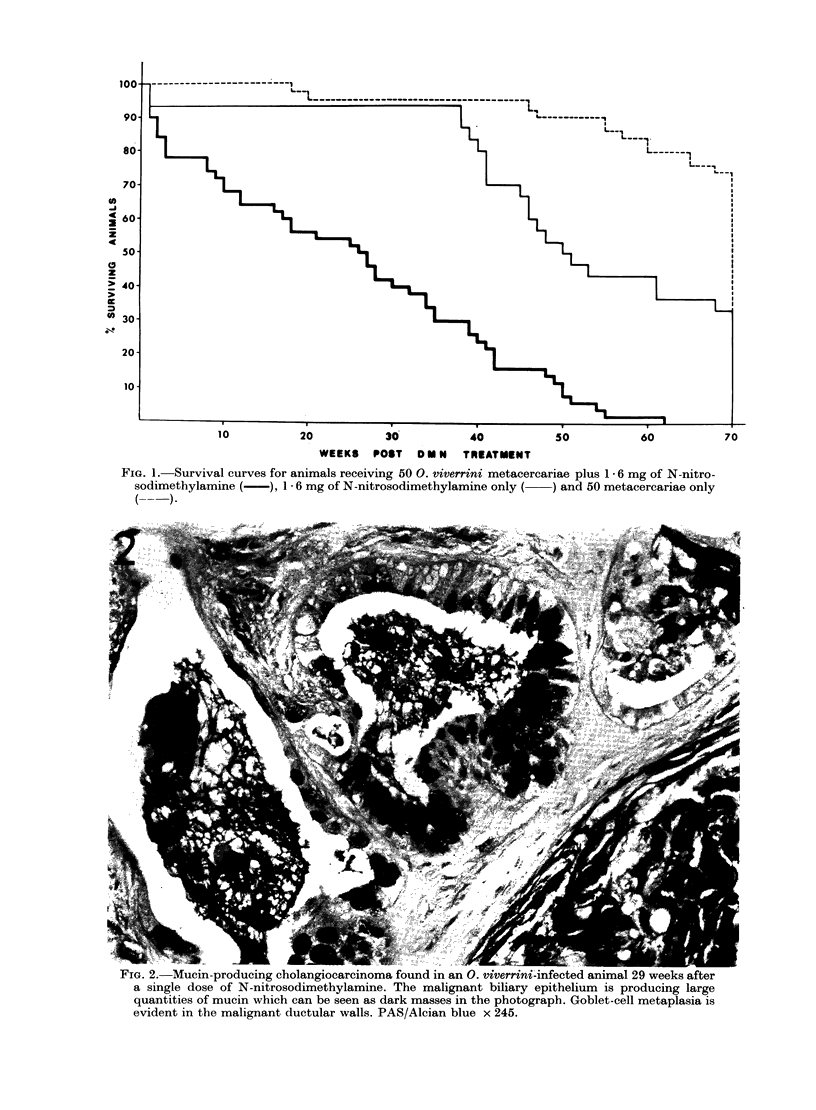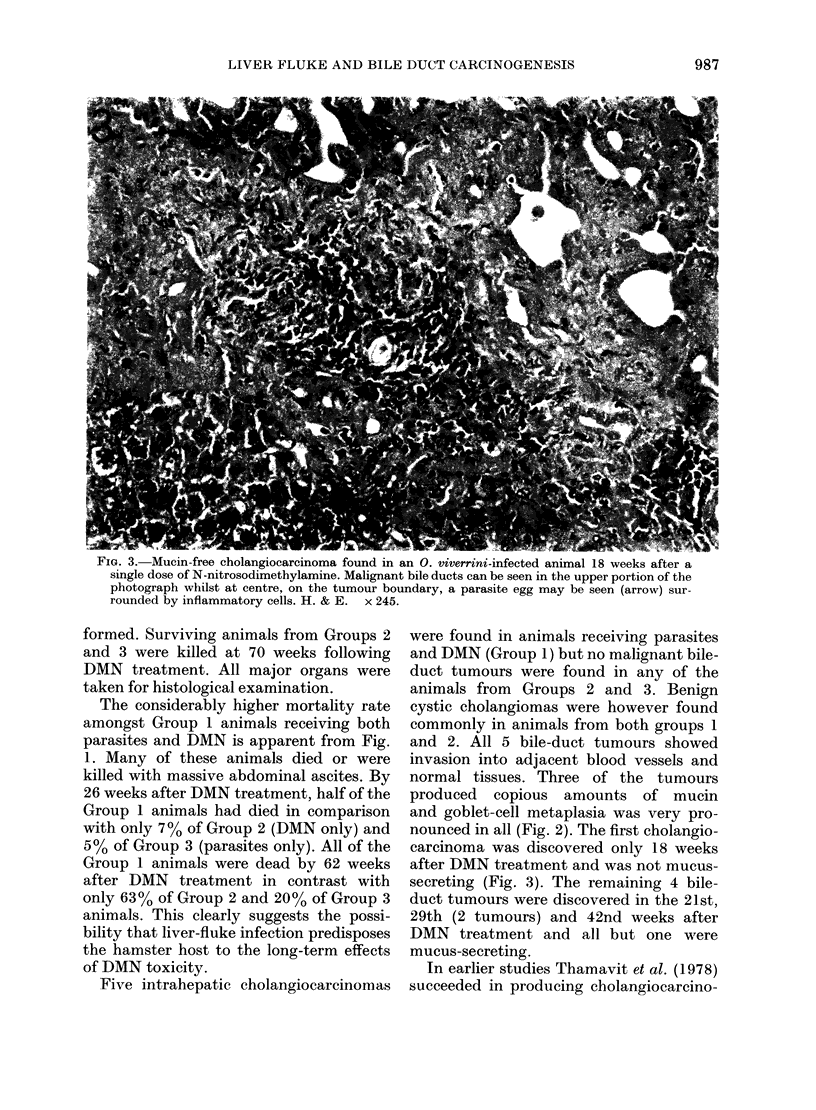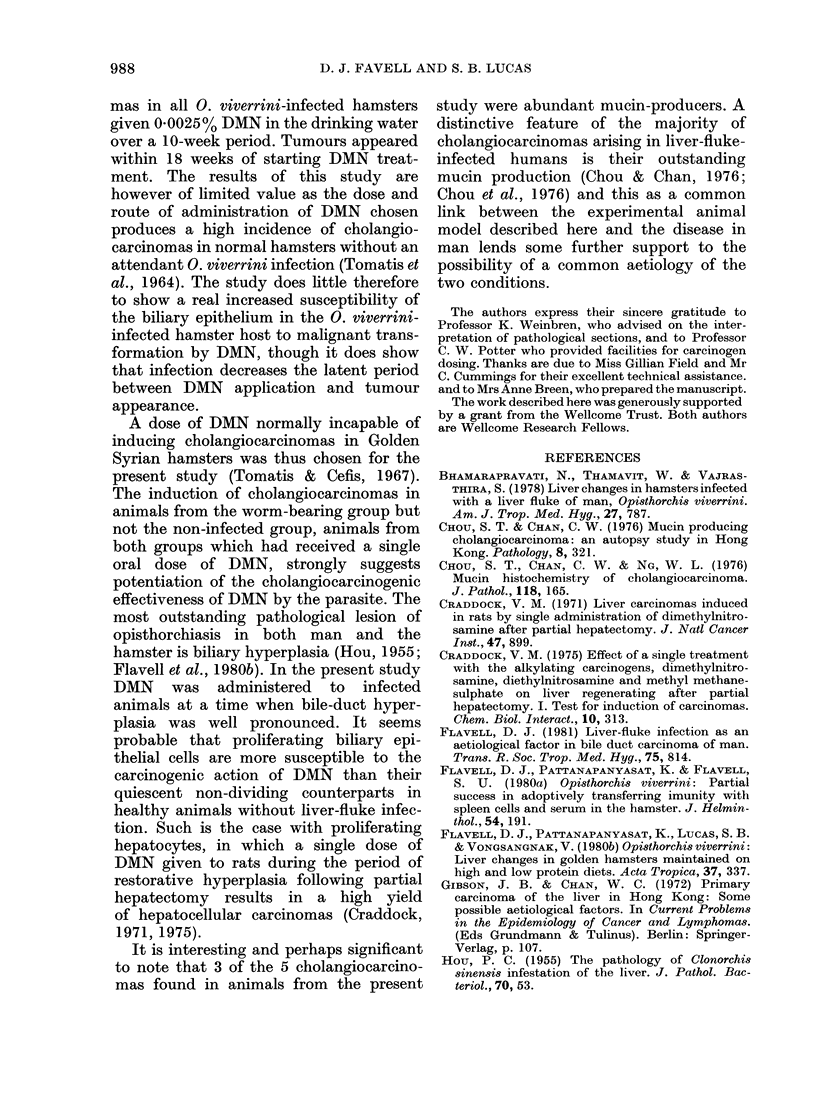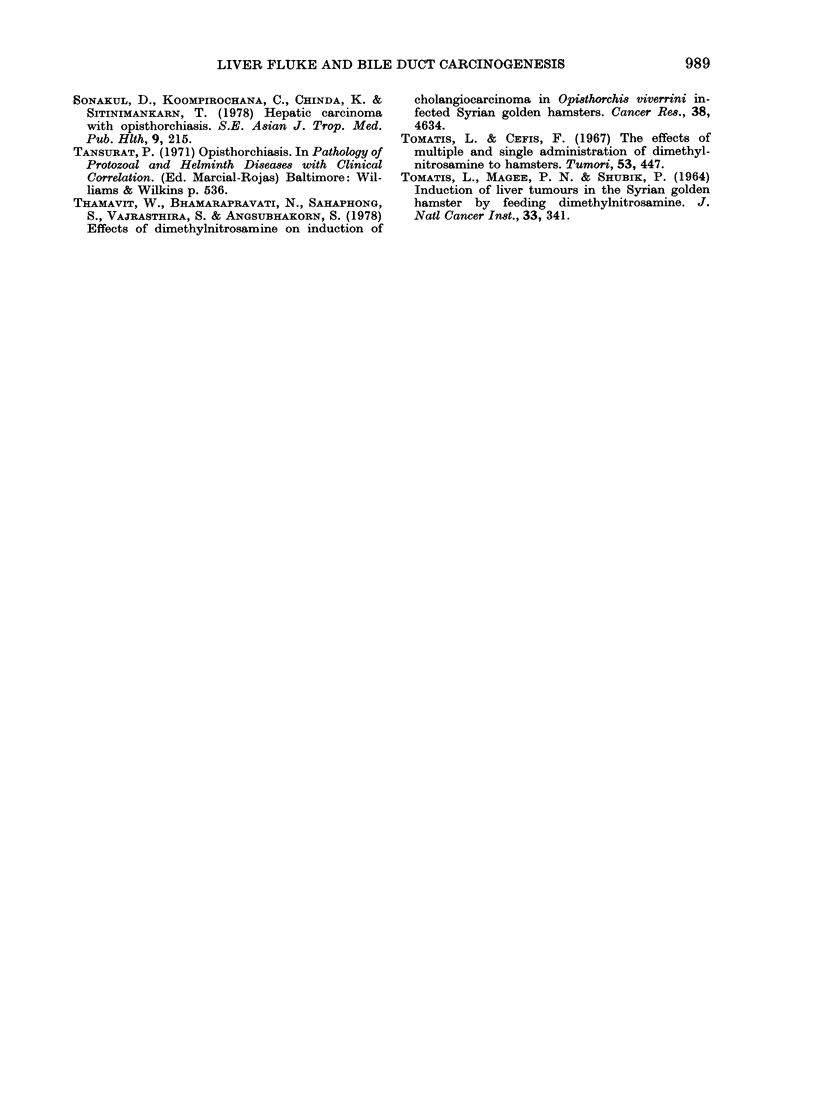# Potentiation by the human liver fluke, Opisthorchis viverrini, of the carcinogenic action of N-nitrosodimethylamine upon the biliary epithelium of the hamster.

**DOI:** 10.1038/bjc.1982.313

**Published:** 1982-12

**Authors:** D. J. Flavell, S. B. Lucas

## Abstract

**Images:**


*Br. J. Cancer* (1982) **46**, 985

***Short Communication***

### POTENTIATION BY THE HUMAN LIVER FLUKE, *OPISTHORCHIS VIVERRINI*, OF THE CARCINOGENIC ACTION OF N-NITROSODIMETHYLAMINE UPON THE BILIARY EPITHELIUM OF THE HAMSTER

D. J. FLAVELL AND S. B. LUCAS*

*From the Department of Medical Heminthology, London School of Hygiene and Tropical Medicine, Winches Farm Field Station, 395 Hatfield Road, St Albans, Herts AL4 0XQ and *Department of Histopathology, St Thomas' Hospital, London SE1 7EH*

Received 5 July 1982 Accepted 3 September 1982

A CAUSAL RELATIONSHIP between infection with the liver fluke, *Opisthorchis viverrini*, and intrahepatic bile-duct carcinoma (cholangiocarcinoma) in man is strongly suspected (Sonakul *et al.*, 1978; Flavell, 1981) though not yet conclusively proven. The liver pathology of opisthorchiasis has been adequately described in man (Tansurat, 1971) and well studied in the hamster (Bhamarapravati *et al.*, 1978; Flavell *et al.*, 1980*b*), though in our experience life-time infections in golden Syrian hamsters do not result in the production of bile-duct tumours.

The hypothesis has been presented that liver-fluke infection of man renders the biliary epithelium more susceptible to malignant transformation by chemical or other exogenous carcinogenic stimuli (Flavell, 1981; Gibson & Chan, 1972; Thamavit *et al.*, 1978). In the study described here we chose to investigate the influence of an experimental *O. viverrini* infection in Golden Syrian hamsters on the pattern of appearance of primary liver tumours following a single treatment with the carcinogen N-nitrosodimethylamine (DMN) at a dose normally ineffective at inducing bile-duct cancer in this animal species.

Three groups of Golden Syrian hamsters were treated as follows:

Group 1. 50 *O. viverrini* metacercariae + 1·6 mg. DMN (50 animals)
Group 2. 1·6 mg.DMN only (30 animals)
Group 3. 50 *O. viverrini* metacercariae only (50 animals)

Metacercariae obtained from naturally-infected Cyprinoid fish were administered to animals intragastrically as described previously (Flavell *et al.*, 1980*b*). Forty-one days after infection, a single oral dose of 1·6 mg. DMN was given to group 1 and 2 animals *via* a dosing needle. At this time after infection, parasites have matured and begun egg production in the extra- and intra-hepatic bile ducts (Flavell, unpublished). Moreover, we (Flavell *et al.*, 1980*b*) and others (Bhamarapravati *et al.*, 1978) have shown that biliary hyperplasia starts within the first 2 weeks of infection in the hamster and that by 41 days after infection biliary epithelial proliferation, particularly of the second order bile ducts where parasites preferentially reside, is well established.

To confirm that infections had been established in all animals from Groups 1 and 3, faecal pellets were collected from each animal just before administration of DMN, and screened for the presence of eggs by a previously described method (Flavell *et al.*, 1980*a*). It was thus established that experimental infections had been successfully established in all animals given metacercariae. Animals were left until natural death or killed when moribund and full post mortems per-

FIG. 1.—Survival curves for animals receiving 50 *O. viverrini* metacercariae plus 1·6 mg of N-nitrosodimethylamine (**——**), 1·6 mg of N-nitrosodimethylamine only (——) and 50 metacercariae only (– – –).

FIG. 2.—Mucin-producing cholangiocarcinoma found in an *O. viverrini*-infected animal 29 weeks after a single dose of N-nitrosodimethylamine. The malignant biliary epithelium is producing large quantities of mucin which can be seen as dark masses in the photograph. Goblet-cell metaplasia is evident in the malignant ductular walls. PAS/Alcian blue × 245.

FIG. 3.—Mucin-free cholangiocarcinoma found in an *O. viverrini*-infected animal 18 weeks after a single dose of N-nitrosodimethylamine. Malignant bile ducts can be seen in the upper portion of the photograph whilst at centre, on the tumour boundary, a parasite egg may be seen (arrow) surrounded by inflammatory cells. H. & E. × 245.

formed. Surviving animals from Groups 2 and 3 were killed at 70 weeks following DMN treatment. All major organs were taken for histological examination.

The considerably higher mortality rate amongst Group 1 animals receiving both parasites and DMN is apparent from Fig. 1. Many of these animals died or were killed with massive abdominal ascites. By 26 weeks after DMN treatment, half of the Group 1 animals had died in comparison with only 7% of Group 2 (DMN only) and 5% of Group 3 (parasites only). All of the Group 1 animals were dead by 62 weeks after DMN treatment in contrast with only 63% of Group 2 and 20% of Group 3 animals. This clearly suggests the possibility that liver-fluke infection predisposes the hamster host to the long-term effects of DMN toxicity.

Five intrahepatic cholangiocarcinomas were found in animals receiving parasites and DMN (Group 1) but no malignant bile-duct tumours were found in any of the animals from Groups 2 and 3. Benign cystic cholangiomas were however found commonly in animals from both groups 1 and 2. All 5 bile-duct tumours showed invasion into adjacent blood vessels and normal tissues. Three of the tumours produced copious amounts of mucin and goblet-cell metaplasia was very pronounced in all (Fig. 2). The first cholangiocarcinoma was discovered only 18 weeks after DMN treatment and was not mucus-secreting (Fig. 3). The remaining 4 bile-duct tumours were discovered in the 21st, 29th (2 tumours) and 42nd weeks after DMN treatment and all but one were mucus-secreting.

In earlier studies Thamavit *et al.* (1978) succeeded in producing cholangiocarcinomas in all *O. viverrini*-infected hamsters given 0·0025% DMN in the drinking water over a 10-week period. Tumours appeared within 18 weeks of starting DMN treatment. The results of this study are however of limited value as the dose and route of administration of DMN chosen produces a high incidence of cholangiocarcinomas in normal hamsters without an attendant *O. viverrini* infection (Tomatis *et al.*, 1964). The study does little therefore to show a real increased susceptibility of the biliary epithelium in the *O. viverrini*-infected hamster host to malignant transformation by DMN, though it does show that infection decreases the latent period between DMN application and tumour appearance.

A dose of DMN normally incapable of inducing cholangiocarcinomas in Golden Syrian hamsters was thus chosen for the present study (Tomatis & Cefis, 1967). The induction of cholangiocarcinomas in animals from the worm-bearing group but not the non-infected group, animals from both groups which had received a single oral dose of DMN, strongly suggests potentiation of the cholangiocarcinogenic effectiveness of DMN by the parasite. The most outstanding pathological lesion of opisthorchiasis in both man and the hamster is biliary hyperplasia (Hou, 1955; Flavell *et al.*, 1980*b*). In the present study DMN was administered to infected animals at a time when bile-duct hyperplasia was well pronounced. It seems probable that proliferating biliary epithelial cells are more susceptible to the carcinogenic action of DMN than their quiescent non-dividing counterparts in healthy animals without liver-fluke infection. Such is the case with proliferating hepatocytes, in which a single dose of DMN given to rats during the period of restorative hyperplasia following partial hepatectomy results in a high yield of hepatocellular carcinomas (Craddock, 1971, 1975).

It is interesting and perhaps significant to note that 3 of the 5 cholangiocarcinomas found in animals from the present study were abundant mucin-producers. A distinctive feature of the majority of cholangiocarcinomas arising in liver-fluke-infected humans is their outstanding mucin production (Chou & Chan, 1976; Chou *et al.*, 1976) and this as a common link between the experimental animal model described here and the disease in man lends some further support to the possibility of a common aetiology of the two conditions.

The authors express their sincere gratitude to Professor K. Weinbren, who advised on the interpretation of pathological sections, and to Professor C. W. Potter who provided facilities for carcinogen dosing. Thanks are due to Miss Gillian Field and Mr C. Cummings for their excellent technical assistance. and to Mrs Anne Breen, who prepared the manuscript.

The work described here was generously supported by a grant from the Wellcome Trust. Both authors are Wellcome Research Fellows.

### REFERENCES

Bhamarapravati, N., Thamavit, W. & Vajrasthira, S. (1978) Liver changes in hamsters infected with a liver fluke of man, *Opisthorchis viverrini*. *Am. J. Trop. Med. Hyg.*, **27**, 787.

Chou, S. T. & Chan, C. W. (1976) Mucin producing cholangiocarcinoma: an autopsy study in Hong Kong. *Pathology*, **8**, 321.

Chou, S. T., Chan, C. W. & Ng, W. L. (1976) Mucin histochemistry of cholangiocarcinoma. *J. Pathol.*, **118**, 165.

Craddock, V. M. (1971) Liver carcinomas induced in rats by single administration of dimethylnitrosamine after partial hepatectomy. *J. Natl Cancer Inst.*, **47**, 899.

Craddock, V. M. (1975) Effect of a single treatment with the alkylating carcinogens, dimethylnitrosamine, diethylnitrosamine and methyl methanesulphate on liver regenerating after partial hepatectomy. I. Test for induction of carcinomas. *Chem. Biol. Interact.*, **10**, 313.

Flavell, D. J. (1981) Liver-fluke infection as an aetiological factor in bile duct carcinoma of man. *Trans. R. Soc. Trop. Med. Hyg.*, **75**, 814.

Flavell, D. J., Pattanapanyasat, K. & Flavell, S. U. (1980*a*) *Opisthorchis viverrini*: Partial success in adoptively transferring imunity with spleen cells and serum in the hamster. *J. Helminthol.*, **54**, 191.

Flavell, D. J., Pattanapanyasat, K., Lucas, S. B. & Vongsangnak, V. (1980*b*) *Opisthorchis viverrini*: Liver changes in golden hamsters maintained on high and low protein diets. *Acta Tropica*, **37**, 337.

Gibson, J. B. & Chan, W. C. (1972) Primary carcinoma of the liver in Hong Kong: Some possible aetiological factors. In *Current Problems in the Epidemiology of Cancer and Lymphomas.* (Eds Grundmann & Tulinus). Berlin: Springer-Verlag, p. 107.

Hou, P. C. (1955) The pathology of *Clonorchis sinensis* infestation of the liver. *J. Pathol. Bacteriol.*, **70**, 53.

SONAKUL, D., KOOMPIROCHANA, C., CHINDA, K. & SITINIMANKARN, T. (1978) Hepatic carcinoma with opisthorchiasis. *S.E. Asian J. Trop. Med. Pub. Hlth*, **9**, 215.

TANSURAT, P. (1971) Opisthorchiasis. In *Pathology of Protozoal and Helminth Diseases with Clinical Correlation.* (Ed. Marcial-Rojas) Baltimore: Williams & Wilkins p. 536.

THAMAVIT, W., BHAMARAPRAVATI, N., SAHAPHONG, S., VAJRASTHIRA, S. & ANGSUBHAKORN, S. (1978) Effects of dimethylnitrosamine on induction of cholangiocarcinoma in *Opisthorchis viverrini* infected Syrian golden hamsters. *Cancer Res.*, **38**, 4634.

TOMATIS, L. & CEFIS, F. (1967) The effects of multiple and single administration of dimethylnitrosamine to hamsters. *Tumori*, **53**, 447.

TOMATIS, L., MAGEE, P. N. & SHUBIK, P. (1964) Induction of liver tumours in the Syrian golden hamster by feeding dimethylnitrosamine. *J. Natl Cancer Inst.*, **33**, 341.